# The effect of ivermectin alone and in combination with cobicistat or elacridar in experimental *Schistosoma mansoni* infection in mice

**DOI:** 10.1038/s41598-021-84009-y

**Published:** 2021-02-24

**Authors:** Belén Vicente, Julio López-Abán, Juliane Chaccour, Juan Hernández-Goenaga, Patricia Nicolas, Pedro Fernández-Soto, Antonio Muro, Carlos Chaccour

**Affiliations:** 1 Infectious and Tropical Diseases Group (e-INTRO), IBSAL-CIETUS (Biomedical Research Institute of Salamanca-Research Centre for Tropical Diseases at the University of Salamanca), Faculty of Pharmacy, University of Salamanca, Salamanca, Spain; 2 Clinica Universidad de Navarra, Pamplona, Spain; 3 ISGlobal, Barcelona Institute for Global Health, Hospital Clínic, Universitat de Barcelona, Rosello 132, 5ª 2ª, 08036 Barcelona, Spain; 4 Ifakara Health Institute, Ifakara, 67501 United Republic of Tanzania

**Keywords:** Drug discovery, Parasitology

## Abstract

*Schistosoma mansoni* is less susceptible to the antiparasitic drug ivermectin than other helminths. By inhibiting the P-glycoprotein or cytochrome P450 3A in mice host or parasites in a murine model, we aimed at increasing the sensitivity of *S. mansoni* to the drug and thus preventing infection. We assigned 124 BALB/c mice to no treatment, treatment with ivermectin only or a combination of ivermectin with either cobicistat or elacridar once daily for three days before infecting them with 150 *S. mansoni* cercariae each. The assignment was done by batches without an explicit randomization code. Toxicity was monitored. At eight weeks post-infection, mice were euthanized. We determined number of eggs in intestine and liver, adult worms in portal and mesenteric veins. Disease was assessed by counting granulomas/cm^2^ of liver and studying organ weight indices and total weight. IgG levels in serum were also considered. No difference between groups treated with ivermectin only or in combination with cobicistat or elacridar compared with untreated, infected controls. Most mice treated with ivermectin and elacridar suffered severe neurological toxicity. In conclusion, systemic treatment with ivermectin, even in the presence of pharmacological inhibition of P-glycoprotein or cytochrome P450 3A, did not result in effective prophylaxis for *S. mansoni* infection in an experimental murine model.

## Introduction

Progress in the control or elimination of schistosomiasis must be approached from different angles: accurate, fast and cheap diagnosis; affordable, safe and effective treatment; and well-established prevention and control strategies. WHO has recognized the need to identify new compounds as an alternative to praziquantel, the single therapeutic agent in use today. Although praziquantel remains effective, concerns arise about potential drug resistance stemming from its continuous use in mass drug administration campaigns in endemic areas^1,2^. Different artemisinin-derived compounds have been developed and tested alone or combined with praziquantel^3^. Mefloquine has also been used, with good results in experimental models^4^, and edelfosine alone or in combination with praziquantel, with good results in reducing granulomatous inflammation both in vitro and in vivo^5,6^.

Ivermectin is a widely used antiparasitic drug^7^. It is the first-line treatment for strongyloidiasis, scabies and onchocerciasis, part of the regimen for lymphatic filariasis (LF) and has demonstrated efficacy against other soil-transmitted helminths^8^. In the mouse model of schistosome infection, adult worm load can be reduced by very high doses of ivermectin^9^, as it appears that *Schistosoma mansoni* is much less susceptible to ivermectin than other helminths^10^. It is unknown whether *S. mansoni* cercariae differ in sensitivity to ivermectin, to date no study has described a prophylactic model.

Decreased susceptibility to ivermectin can be brought about by ATP-binding cassette (ABC) transport proteins and their interaction with cytochrome P450 (CYP) 3A^11,12^. We hypothesize that the combination of ivermectin and elacridar, a P-glycoprotein inhibitor, or cobicistat, a CYP3A inhibitor, could increase the susceptibility of *S. mansoni* invading schistosomula to ivermectin. The objective of this work was to evaluate the capacity of ivermectin alone and in combination with elacridar or cobicistat to prevent *S. mansoni* infection in an experimental model using BALB/c mice.

## Materials and methods

### Parasites and animals

To maintain the *Schistosoma mansoni* (LE strain) cycle, freshwater snails *Biomphalaria glabrata* were used as intermediate hosts, and CD1 mice as definitive hosts. Snails of 4–8 mm in diameter were infected with seven miracidia each. They were kept in 25 °C water for 30 days until the emission of furcocercariae was induced with light and a temperature of 26 °C for 2 h. Triplicate counts were done to obtain a dose of 150 cercariae in 0.7–1.2 ml of chlorine-free water.

For the infection experiment, 124 SPF BALB/c mice (Charles River, Lyon, France) with a weight of 18.5–21.3 g and an age of seven weeks were used. The animals were kept in a controlled temperature and humidity environment with a 12:12 h light:dark cycle. They were supplied with water and food ad libitum in the facilities of the Animal Experimentation Service of the University of Salamanca according to the current Spanish legislation on animal experimentation (L32/2007, L6/2013 and RD 53/2013) and the transposition of the rules of the European Union (Di 2010/63/CE). All experiments with animals were approved by the Bioethics Committee of the University of Salamanca (Registration number CBE-225). Humane endpoints were applied when an evidence of severe pain, excessive distress, suffering or an impending death was observable in any of the animals, which were then euthanized. All mice were euthanized at the end of the experiment by intraperitoneal injection of sodium pentobarbital in PBS (100 mg/kg). The status of all mice was checked daily using a composite score including vitality, secretions, fur quality, mobility, dyspnea, ascites, neurological signs, and ability to ingest water or food.

All methods were carried out in accordance with relevant guidelines and regulations. All animal handling and methods complied with the ARRIVE guidelines.

### Experimental design

**Figure 1:**
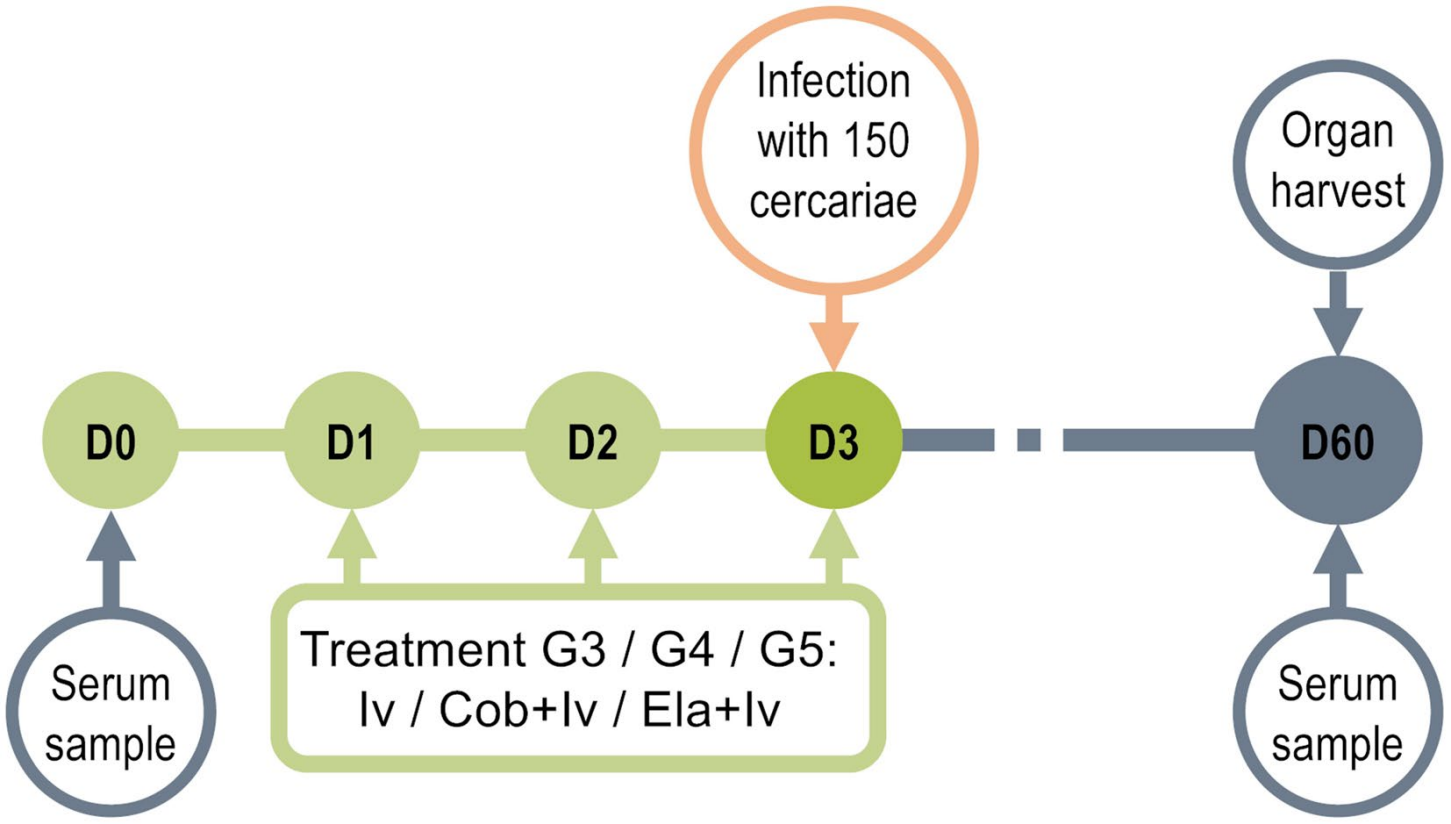
Study timeline. Ivermectin or ivermectin and cobicistat or ivermectin and elacridar were administered daily for three days before infecting BALB/c mice with 150 cercariae of *S. mansoni*. After 8 weeks of infection, parasite load in organs and IgG production against SoSmAWA antigen were examined. Treatment tolerance was monitored throughout.

We used the BALB/c mouse model of infection by *Schistosoma mansoni* established in the IBSAL-CIETUS of the University of Salamanca, Spain. Mice were divided into five experimental groups: untreated uninfected (G1 Untr, n = 9), infected (G2 Inf, n = 45), treated with 1000 μg/kg of ivermectin daily for three days by oral catheter before infection (G3 Iv, n = 30), treated with 1000 μg/kg of ivermectin and 25 mg/kg of cobicistat daily for three days by oral catheter before infection (G4 Iv + Co, n = 30), and treated with 1000 μg/kg of ivermectin and 2.5 mg/kg of elacridar daily for three days by oral catheter before infection (G5 Iv + El, n = 10) (Fig. 1). In G4 and G5, ivermectin was administered 2 h after cobicistat and elacridar.

After each drug administration, the behavior of the animals was observed for potential signs of toxicity (nervous, neuromuscular, digestive). On the third day of treatment and 4 h after administering the ivermectin dose, the animals in group 2, 3, 4 and 5 were infected with 150 cercariae of *S. mansoni* each. The infection was performed after anesthesia with a mixture of ketamine (50 mg/kg), diazepam (5 mg/kg) and atropine (1 mg/kg), in a final volume of 100 µl by intraperitoneal injection in order to immobilize the animals. They were placed supine, a plastic ring was placed on the shaved abdomen, attached with adhesive tape, and the water solution was applied with cercariae. After 45 min the rings were removed from the abdomen of the mice and the water dried. Once the mice recovered mobility after sedation, the mice were returned to their corresponding cages.

The animals were weighed and had blood samples taken before starting the experiment, before infection and before the necropsies. Eight weeks post-infection, the mice were euthanized. Serum was obtained from the blood samples by centrifugation at 8000 rpm at 4 °C for 8 min. Samples were stored at − 20 °C until use.

To recover adult worms from the portal vein and mesenteric veins, livers were sectioned and perfused with a 0.9% saline solution with heparin through the left ventricle. Adults worms were recovered with tweezers and the number of *Schistosoma* couples and single male and female worms was counted.

A part of the intestine and part of the liver of each mouse were also separated and weighed. Subsequently, they were digested to determine the egg load. For this, both liver and intestine samples were incubated at 37 °C for 24 h in 5% KOH while stirring. The digested samples were centrifuged at 800*g* for 5 min and the supernatant was removed, leaving 5 ml of concentrated egg solution. A McMaster chamber was used to count each sample in triplicate and calculate the number of eggs per gram of intestine or liver. Spleen, bowel and liver indices were obtained from the weights of these organs and the live weight of the mice to determine their degree of inflammation. Photographs were taken where macroscopic lesions of the livers were observed to estimate the number of granulomas per square centimeter. The ImageJ v.1.51k, PowerPoint and Adobe Photoshop CC 2017 programs were used to count granulomas and estimate the affected liver surface.

### ELISA for determination of *S. mansoni* soluble somatic antigen (SoSmAWA)-specific IgG

In order to detect specific antibodies against *S. mansoni* infection, the mouse sera were analyzed by indirect enzyme immunoassay (ELISA), for specific immunoglobulin G (IgG) levels. We used soluble adult somatic antigen from *Schistosoma mansoni* (SoSmAWA) according to Abán et al. (1999)^13^.

We coated 96-well polystyrene plates (Costar 3369, Corning Inc.) with 2.5 µg of SoSmAWA per well in a final volume of 100 µl of carbonate buffer at pH 9.6 and incubated them for 18 h at 4 °C. Plates were washed three times with 200 µl of 1X PBS per well at pH 7.2 and 0.05% Tween (PBS-T) to remove residues of unbound antigen, and blocked with 2% BSA in PBS-T (100 µl per well) at 37 °C for 1 h to avoid nonspecific antibody-binding and washed three times. Serum samples from each mouse were incubated for 1 h at 37 °C at a 1:100 dilution in PBS-T, and washed as in the previous steps. The secondary mouse anti-IgG antibody (Sigma Aldrich, anti-mouse IgG-peroxidase, A5906) was added at a 1:1000 dilution in PBS-T, incubated for 1 h at 37 °C, and washed. The plates were then incubated with o-phenylenediamine dihydrochloride (OPD) as a peroxidase substrate, and H_2_O_2_ as an oxidizing agent in citrate buffer at pH 5.0. The reaction was stopped with 50 µl per well of 3 N H_2_SO_4_. Absorbance was measured at 492 nm in a spectrophotometer (ThermoScientific Multiskan GO 1510, Finland).

### Statistical analysis

Data are presented as means and standard errors of the mean. A Bartlett test was carried out to verify the homogeneity of the data distribution of all the variables. A *t*-unpaired two-tailed ANOVA analysis was done and, if statistically significant, the data was analyzed in a post hoc Fisher’s Least Significant Difference test to determine the existence of significant differences between the study groups. *P*-values below 0.05 were considered significant. A multivariate analysis of main variables was also carried out. For these analyses and work chart, we used Simfit V7.3.0 and Statview V5.0 statistical packages.

## Results

### Effect of Iv and Iv-Co on the weight of BALB/c mice after infection by *S. mansoni*

**Figure 2:**
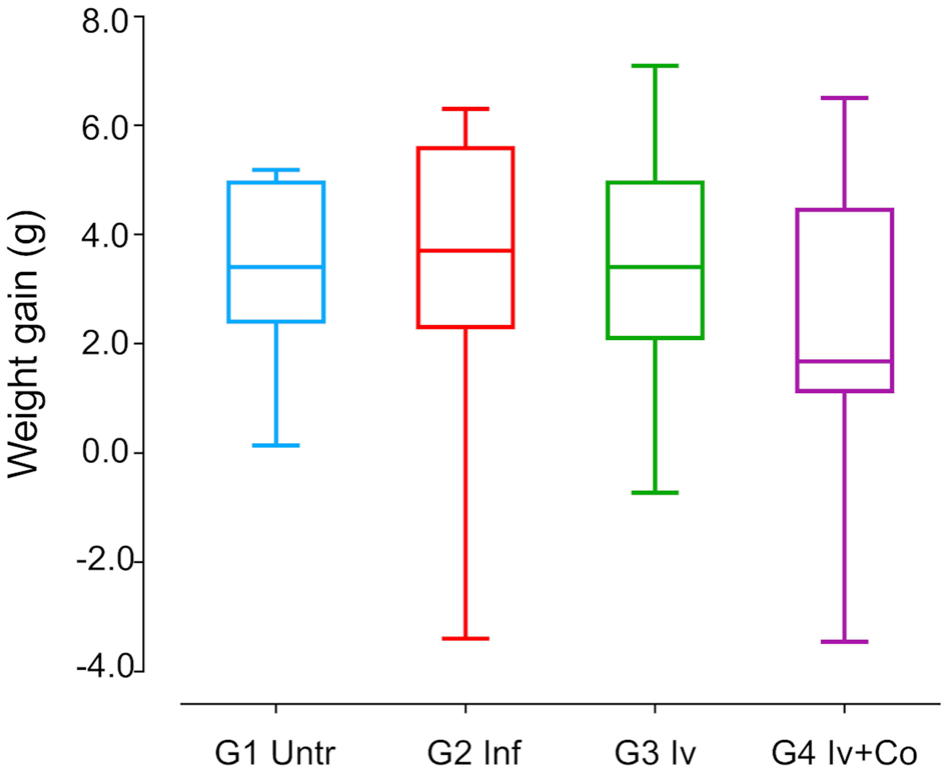
Weight gain throughout experimental period of eight weeks post-infection. Mice treated with ivermectin (Iv) or ivermectin and cobicistat (Iv + Co), infected with 150 cercariae of *S. mansoni* (inf) and control without treatment and infection (Untr).

Weight loss after the administration of a xenobiotic can be an indicator of toxicity in the protocol used. Healthy animals gained an average of 3.2 ± 0.6 g during the experimental period, whereas infected animals gained 3.6 ± 0.3 g. Animals treated with ivermectin after infection gained 3.5 ± 0.5 g, whereas those treated with ivermectin + cobicistat only gained 2.5 ± 0.5 g. These differences were not statistically significant (Fig. 2).

### Neurotoxicity

After administering the combination of ivermectin and elacridar to G5 as detailed in “Materials and methods”, the animals showed seizures, circular movements, and loss of ambulatory capacity. Six animals in the ivermectin and elacridar group died or were euthanized according to the end-point rules of the protocol. Only four out of ten mice survived the administration of the ivermectin and elacridar drug combination. No deaths or signs of neurotoxicity were seen in the group that was administered ivermectin alone or combined with cobicistat.

The four mice surviving in the elacridar and ivermectin group were infected and necropsy performed at eight weeks post infection. In the necropsy, no differences in worms recovered (8.5 ± 3.5 males, 6.0 ± 3.8 females) and egg in tissues were (1992 ± 1082 egg per gram of liver and 1493 ± 801 egg per gram of small intestine) with respect to other groups were seen, no statistical test was performed given the sample size (n = 4).

### Effect of Iv and Iv-Co on worm recovery

**Figure 3:**
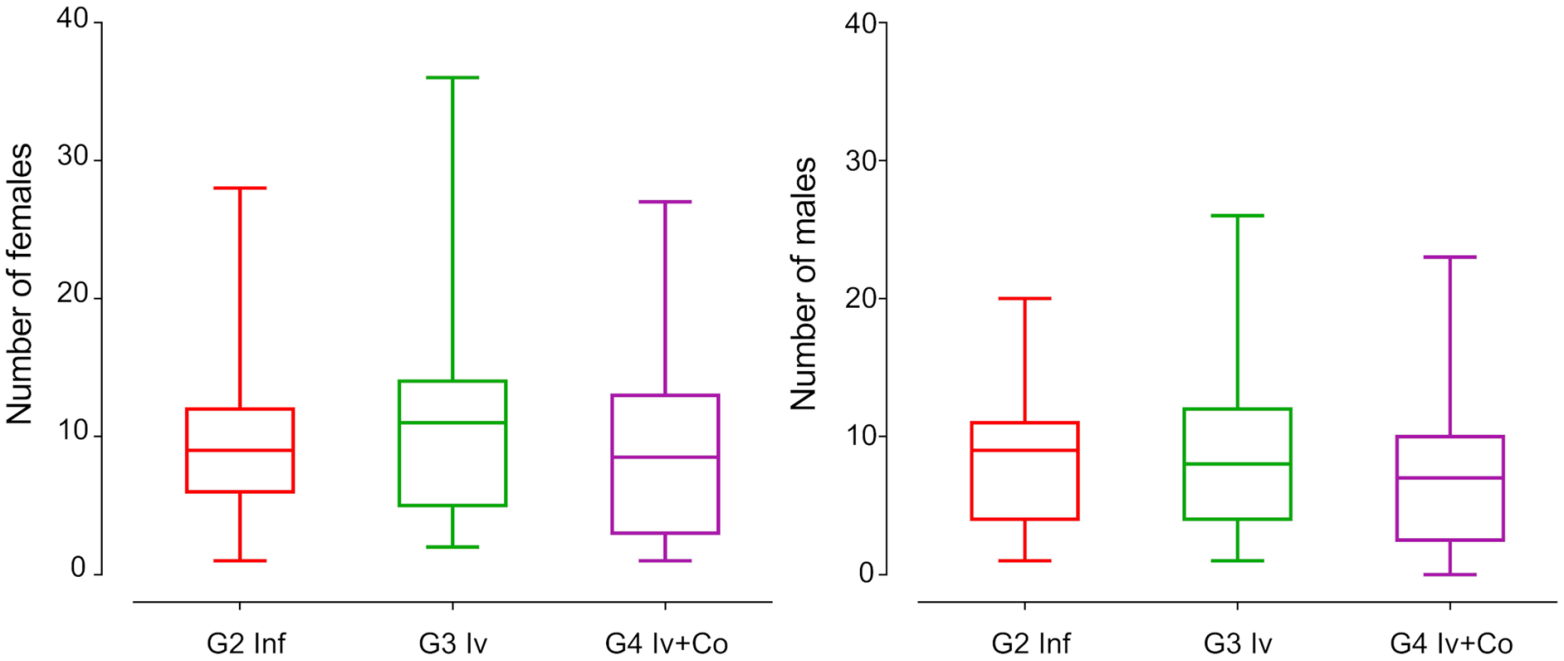
Male and female worm recovery in the necropsy eight weeks post-infection of mice treated with ivermectin (Iv) or ivermectin and cobicistat (Iv + Co), infected with 150 cercariae of *S. mansoni* (inf).

The parasite load was used to estimate the prophylactic capacity of the drug or drug combinations against infection by *S. mansoni*. Mice treated prophylactically with ivermectin showed no significant reduction in the total recovery of adult worms. The animals treated with the combination of ivermectin and cobicistat had a non-significant 8% reduction in worm load compared with the control group of infected mice. A similar situation occurred in the separate recovery of male and female worms (6–12% reduction) (Fig. 3).

### Effect of Iv and Iv-Co on the number of eggs retained in liver and intestine

**Figure 4:**
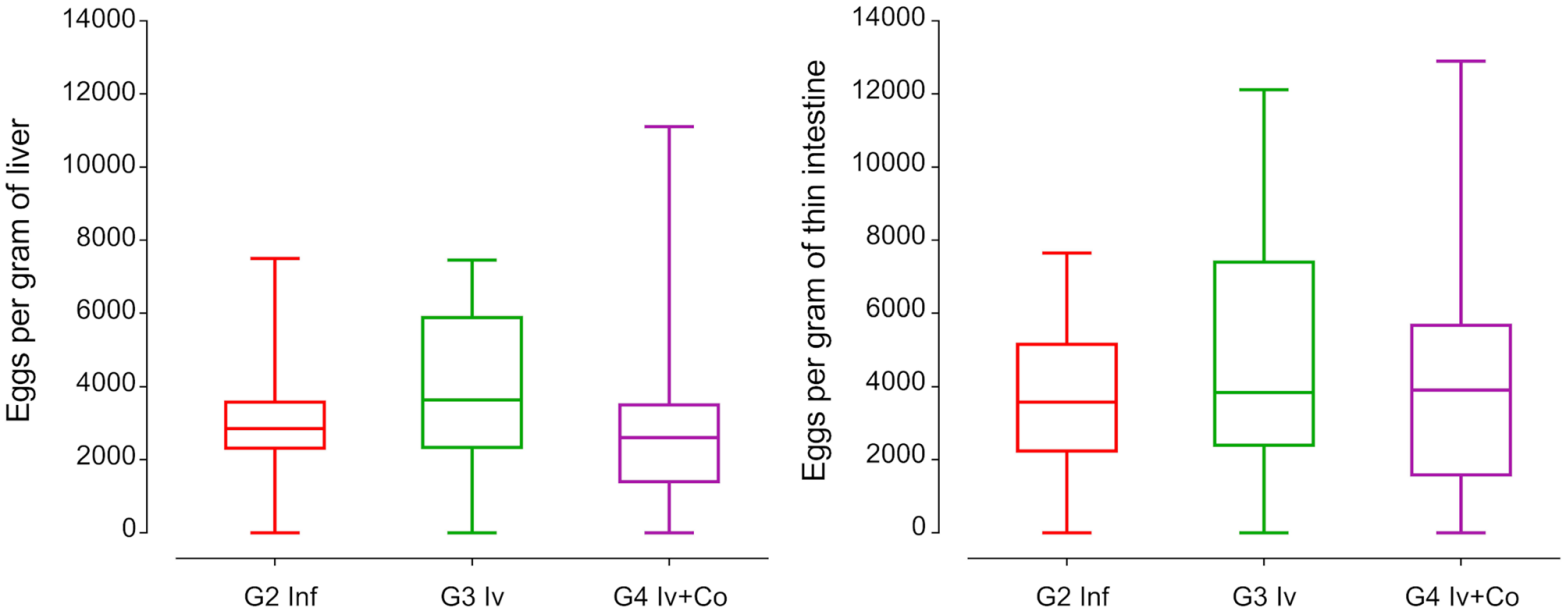
Eggs per gram of liver or thin intestine at the necropsy eight weeks post-infection of mice treated with ivermectin (Iv) or ivermectin and cobicistat (Iv + Co), infected with 150 cercariae of *S. mansoni* (inf).

Schistosome eggs in liver and intestine are the most relevant indicator of the severity of the disease. Mice treated with ivermectin or the combination of ivermectin and cobicistat did not reveal reductions in the number of eggs per gram of liver or intestine compared with the infected control group (Fig. 4).

### Effect of Iv and Iv-Co on S. mansoni granulomas and organ indexes

**Figure 5:**
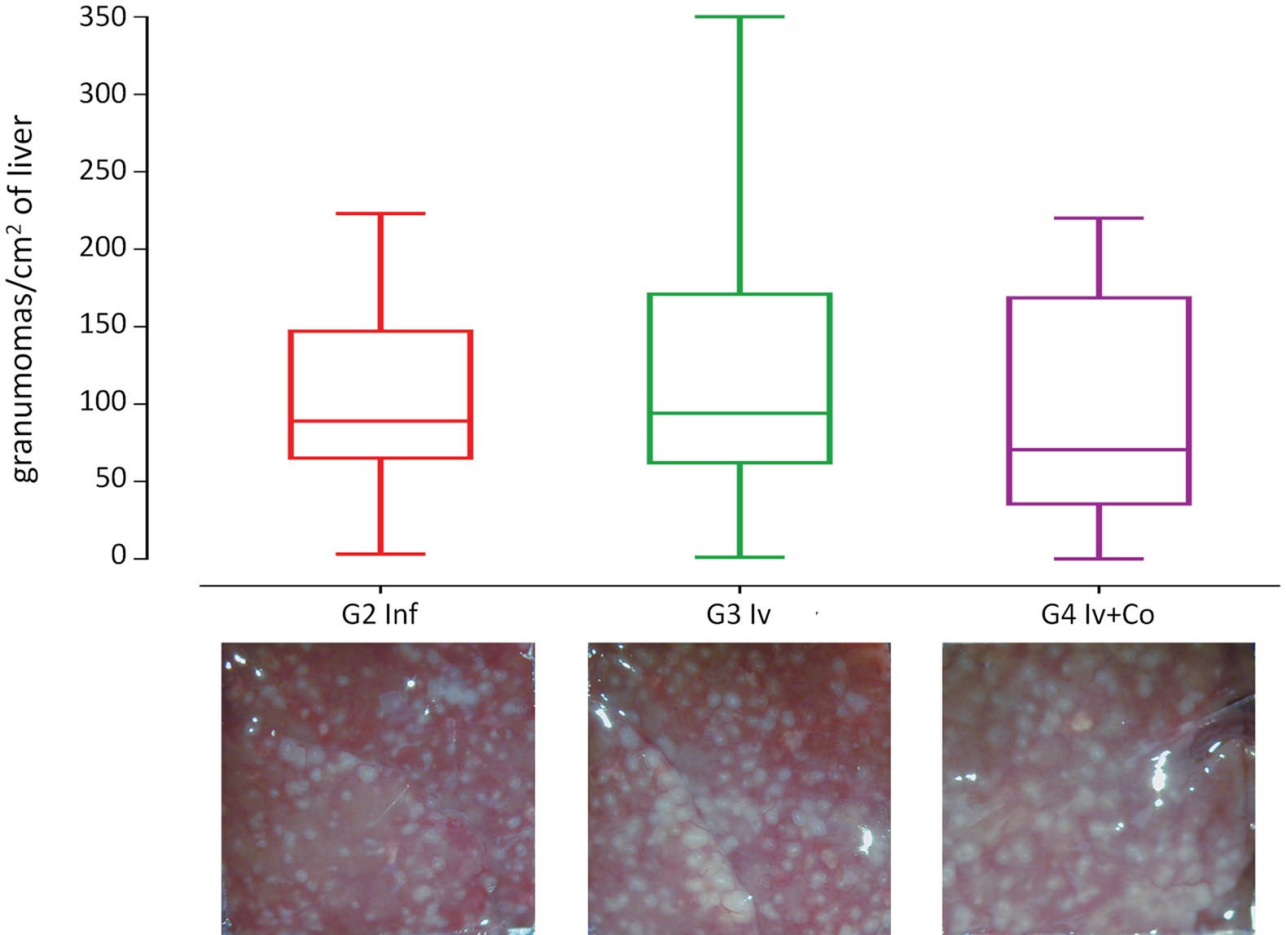
Granulomas in liver surface at the necropsy eight weeks post-infection of mice treated with ivermectin (Iv) or ivermectin and cobicistat (Iv + Co), infected with 150 cercariae of *S. mansoni* (inf).

**Figure 6:**
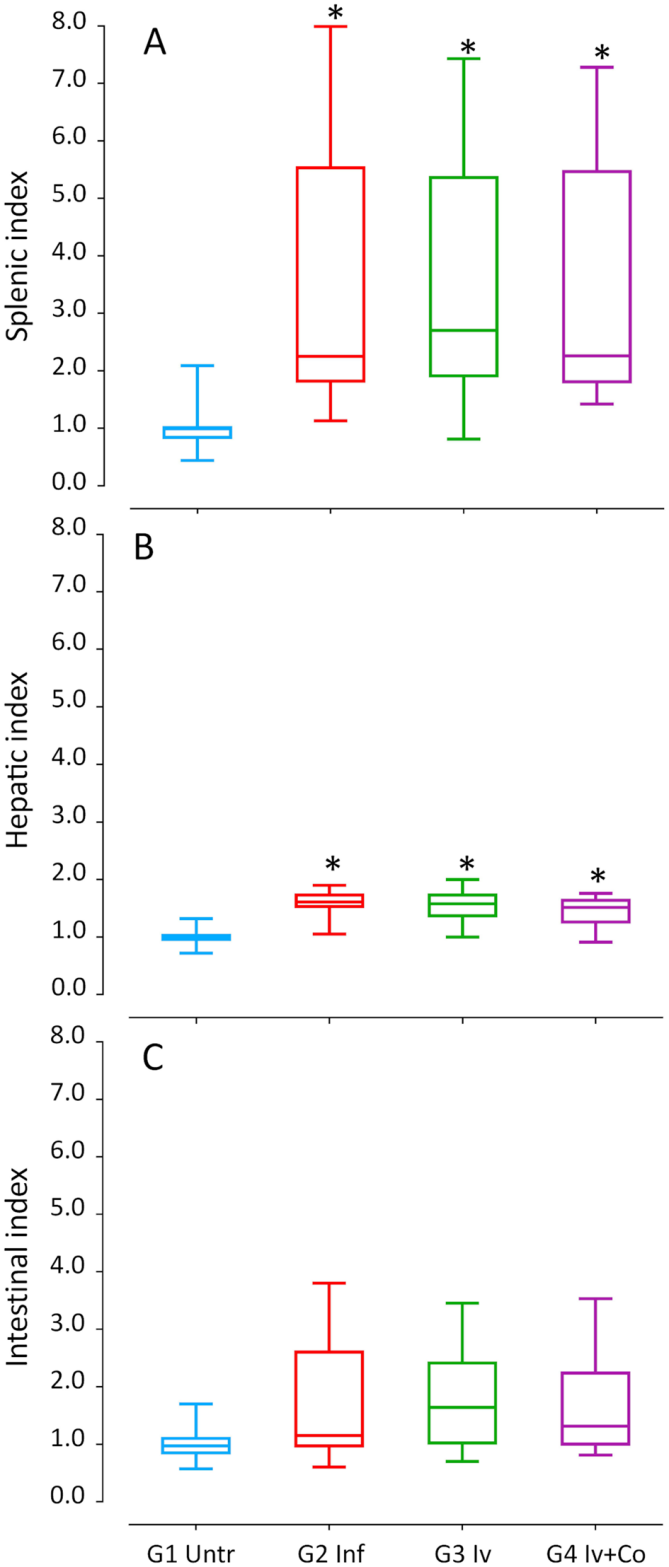
Splenic index (**A**), hepatic index (**B**) and intestinal index (**C**) of mice treated with ivermectin (Iv) and Ivermectin + cobicistat (Iv + Co) and infected with 150 cercariae by *S. mansoni* (Inf) at 8 weeks post-infection. *Statistically significant differences p < 0.05 compared to untreated controls (Untr).

Granuloma formation is an immune response to the presence of eggs in tissues and it is an indicator of severe disease. There was no reduction in granulomas per liver surface in animals that were administered ivermectin When using the combined ivermectin + cobicistat, the reduction was 3.6% but this was not statistically significant when compared with infected mice (Fig. 5). Hepatic and intestinal indices are indicators of the relative inflammation of each of these organs against the eggs trapped. In schistosome infection, hepato-splenomegaly is described as one of the most prominent signs and is present in severe forms of the disease. Infected mice showed a 350–360% increase in spleen weight (ANOVA F_(3.105)_ 4.439, p = 0.006) and a 45–59% increase in liver weight (ANOVA F_(3.105)_ 16.670, p < 0.001) compared with the untreated infected mice. The intestinal index increased by 64–72%, which was not statistically significant (Fig. 6).

### IgG levels against SoSmAWA in infected animals

**Figure 7:**
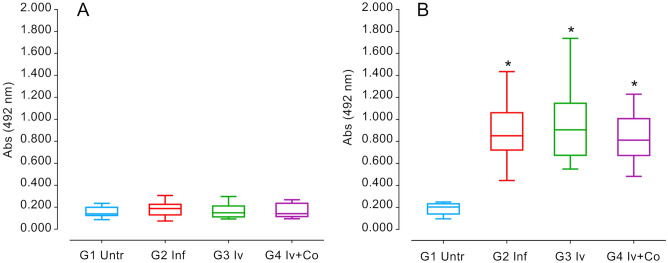
ELISA detection of specific IgG antibodies to SoSmAWA antigen at the beginning of the experiment (**A**) and 8 weeks post-infection (**B**) of mice treated with ivermectin (Iv) and Ivermectin + cobicistat (Iv + Co) and infected with 150 cercariae by *S. mansoni* (Inf). *Statistically significant differences p < 0.05 compared to untreated controls (Untr).

Immunoglobulin levels during infection with *S. mansoni* serve to reveal the status of the infection and the response against the infection of the animals. High levels of specific IgG against the SoSmAWA antigen were observed in all infected groups at eight weeks post-infection. The infected group did not differ significantly from the groups given preventative treatment (Fig. 7).

### Multivariate analysis

**Figure 8:**
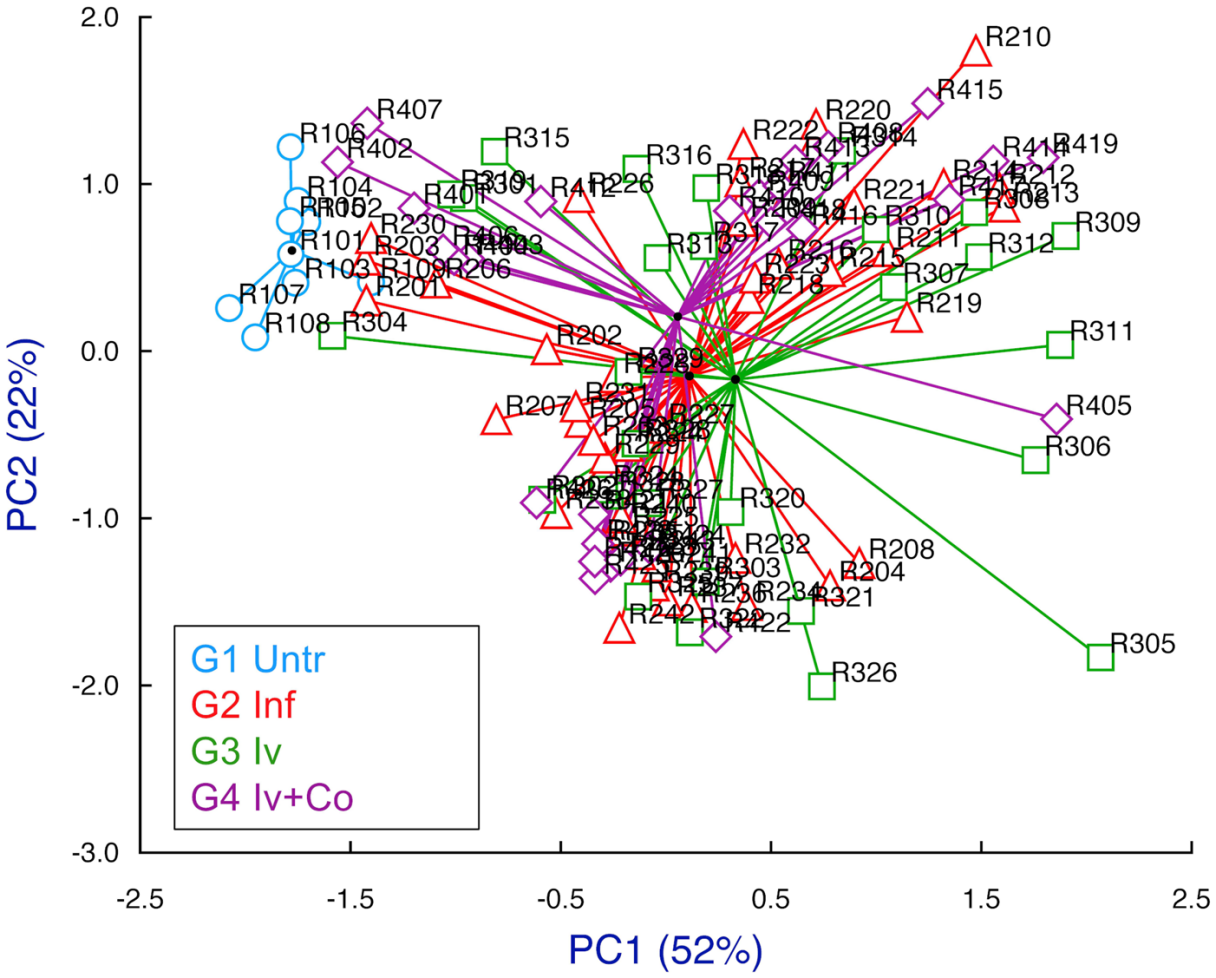
Multivariate study of principal components highlighting each study group and searching their reference centroid at eight weeks post-infection. Groups: mice treated with ivermectin and infected with 150 cercariae of, *S. mansoni* (Iv); treated with ivermectin + cobicistat (Iv + Co) and infected; infected only with 150 cercariae of (Inf): and an untreated and uninfected control group (Untr).

**Figure 9:**
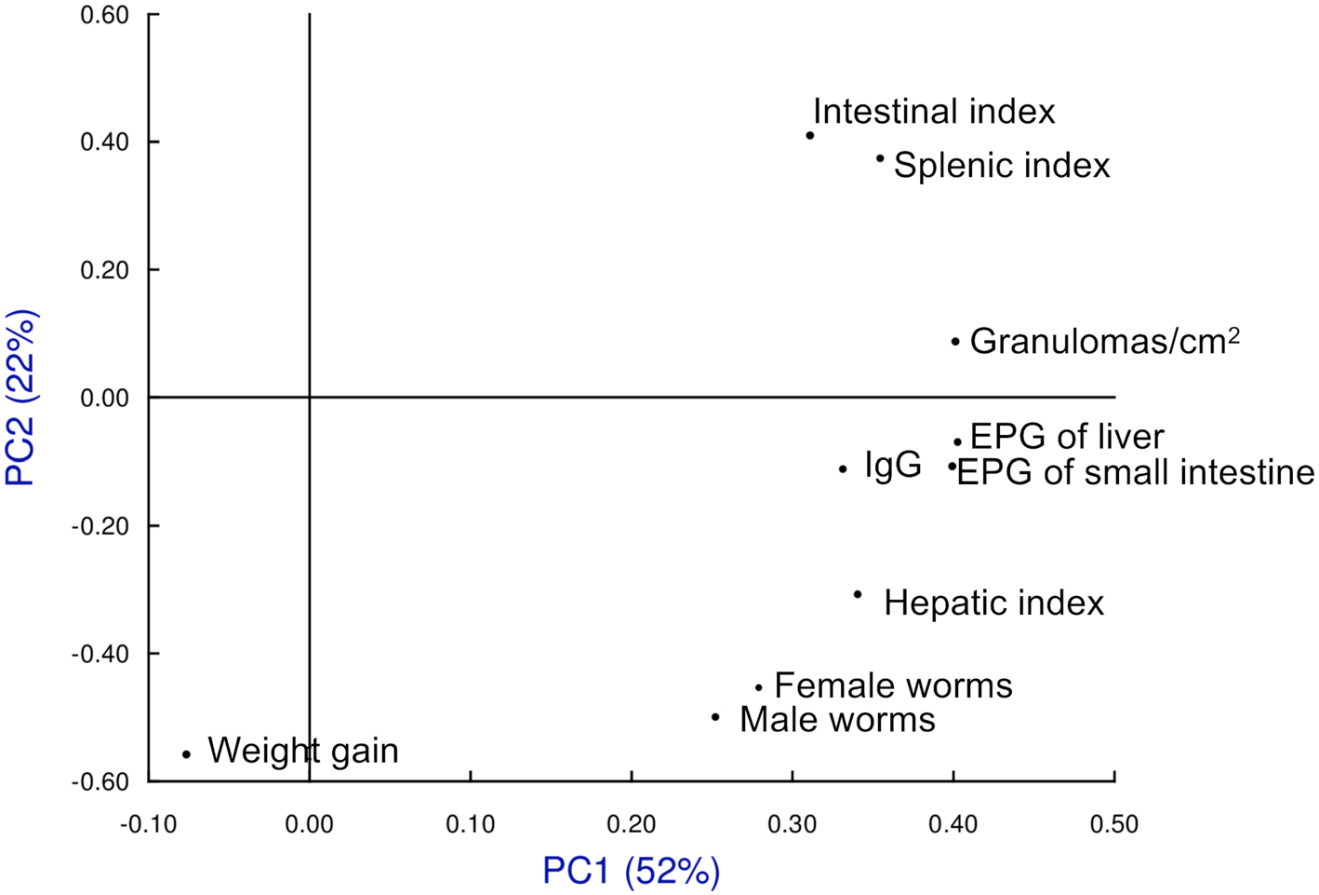
Multivariate study of principal components, loads associated with each of the variables used in the study at eight weeks post-infection. Groups: mice treated with ivermectin and infected with 150 cercariae of, *S. mansoni* (Iv); treated with ivermectin + cobicistat (Iv + Co) and infected; infected only with 150 cercariae of (Inf): and an untreated and uninfected control group (Untr). *EPG* eggs per gram.

We included the following variables for the study of principal components (PC): total recovered worms, eggs in tissue, granulomas in the liver, organ indices, weight variation during the experiment and antibody levels at eight weeks post-infection. Between the principal components 1 and 2 (PC1 and PC2), 74% of the information was collected (Fig. 8). We found no differences between the infected groups previously treated with ivermectin (G3 Iv), ivermectin + cobicistat (G4 Iv-Co) or untreated (G2 Inf). Only the untreated and uninfected group (G1 Untreated) differed (Fig. 5). All variables affected both principal component 1 and 2 (Fig. 9).

### Regulatory compliance

All methods were carried out in accordance with relevant guidelines and regulations.

## Discussion

The discovery of glutamate-mediated signaling in *S. mansoni* raised hopes for new drug targets in this species^14^. Macrocyclic lactones such as ivermectin target the neuronal glutamate-gated chloride channel that is expressed by arthropods, nematodes and trematodes^15^. Schistosomes express these channels, yet the evidence base for the efficacy of ivermectin against *S. mansoni* is inconclusive, which might be explained by a relatively low affinity of the *S. mansoni* glutamate-gated chloride channel for ivermectin^10^. Nevertheless, one study reported a marked reduction in adult worm load after high-dose treatment with ivermectin, which was attributed to the tegumental damage inflicted on the worms^9^. The slight, yet non-significant, reduction in adult worm load observed in our study might indicate a possible dose-dependent effect, as Taman et al*.* used a 25-fold higher dose of ivermectin.

These extremely high doses are contraindicated for systemic human use due to safety concerns. It could be worthwhile exploring a potential alternative approach for targeting the development of schistosome in tissue. We are not aware of data showing differential expression of GluCl channels at different life stages of *S. mansoni*. l-Glutamate-containing neurons have been reported in cercaria that have not been identified in other life stages^16^. It is, however, unknown if ivermectin treatment affects these life stages differently. Using an inhaled formulation against schistosomula stages in the lung might allow for higher concentrations against a developmental stage that is not often targeted^17^.

In *C. elegans*, the susceptibility to ivermectin can be modulated by targeting ABC transporters or the P-gp^18,19^. We previously showed in a pharmaco-enhancement model of mosquitoes feeding on treated pigs that the simultaneous inhibition of cytochrome p450 3A and the P-gp enhanced the effect of ivermectin by two mechanisms: by increasing plasma levels in the pig host and by increasing the susceptibility of the mosquito. This combination resulted in prolonged target insecticidal concentrations of the drug able to kill *Anopheles* mosquitoes^20^. Given the inconclusive evidence regarding the efficacy of ivermectin treatment in *S. mansoni*-infected mice, we hypothesized that P-gp and CYP3A inhibitors may also alter the susceptibility of *S. mansoni* cercariae to ivermectin in a prophylaxis model.

Despite pharmacological inhibition of CYP and P-gp in our study, ivermectin did not have any significant effect on the numbers of *S. mansoni* eggs or adult worms in the mouse nor on the host immune response as evidenced by a lack of differences in IgG levels or granuloma formation. This finding stands in contrast to Kasinathan et al. who showed a significant effect of P-gp inhibitors in combination with praziquantel on both egg numbers and granuloma size in *S. mansoni*-infected mice, and suggested effects on parasite immunomodulatory factors to the host^21^. These differences point to different mechanisms involved in the metabolism of praziquantel and ivermectin.

Ivermectin has an excellent safety profile demonstrated over decades in global mass drug administration campaigns. Its safety is partly explained by the activity of P-gp in the capillaries of blood–brain barrier that excludes ivermectin from the mammalian central nervous system. Only a few cases of encephalopathy were described in rare population genotypes (mdr-1 gene) in Loa loa massive drug administration campaigns^22^.

In our study, the combined use of ivermectin and P-gp inhibitor elacridar led to seizures and disturbances in movement defined as final point criteria in most animals in the treated group. As elacridar disrupts the function of P-gp in the murine blood–brain barrier, elevated ivermectin levels in the brain lead to important neurological toxicity^23,24^. It serves as a reminder that in states with an impaired blood–brain barrier, as is the case in hyperinflammatory states, ivermectin could cause significant neurological toxicity.

A limitation of this study pertains to our choice of model animal. Although ivermectin does not affect physiological parameters in mice, they are one of the rodent species most susceptible to ivermectin toxicity^25^. The data on lethal dose included by Merck in the ivermectin label were determined in murine models^26^. This dose potentially does not represent the true toxicity in larger mammals or humans. The vulnerability of mice to ivermectin toxicity excludes the possibility of using higher doses that might produce more pronounced prophylactic effects against *Schistosoma* infection. Another limitation concerns the standard time span and cercaria dose in which the experiments were conducted to make comparable with other studies. In field conditions people became infected in several times with lower daily doses and measurement time point is difficult to establish. The drug-treated groups were not treated all simultaneously, which might have a small influence on the results.

## Conclusions

Ivermectin did not show prophylactic properties against experimental infection with *S. mansoni* cercariae. Parasite load, granulomatous lesions, or antibody responses in the ivermectin-treated group were comparable to the untreated control group. Combining ivermectin with cobicistat to prevent infection by *S. mansoni* did not result in differences in parasite load, granulomatous lesions, or antibody responses compared to the untreated control group. The combination of ivermectin and elacridar led to severe toxicity.

## Financial support

This study was supported by the University of Navarra with a generous donation from Jeffery and Heart Deal. CCh and PN received salary support from Unitaid through the BOHEMIA grant to ISGlobal. ISGlobal acknowledges support from the Spanish Ministry of Science and Innovation through the “Centro de Excelencia Severo Ochoa 2019–2023” Program (CEX2018-000806-S), and support from the Generalitat de Catalunya through the CERCA Program. USAL acknowledges support from the Instituto de Salud Carlos III, ISCIII, Spain (http://www.isciii.es) under grants: RICET RD16/0027/0018 and PI19/01727; Ministerio de Ciencia e Innovación (MICINN) Spain RTI2018-099474-B-I00 and European Union co-financing by FEDER (Una manera de hacer Europa, Sistema Nacional de Garantía Juvenil, Cofinanciación por FEDER, and Iniciativa de Empleo Juvenil BDNS: 427002).

## Data Availability

All study data is contained within this manuscript and the supplementary material.
